# Alcohol consumption among pregnant women in Northern Tanzania 2000–2010: a registry-based study

**DOI:** 10.1186/s12884-015-0630-0

**Published:** 2015-09-03

**Authors:** Alexander Blaauw Isaksen, Truls Østbye, Blandina Theophil Mmbaga, Anne Kjersti Daltveit

**Affiliations:** Faculty of Medicine and Dentistry, University of Bergen, Bergen, Norway; Department of Global Public Health and Primary Care, University of Bergen, Bergen, Norway; Department of Paediatrics and Child Health, Kilimanjaro Christian Medical Centre and Kilimanjaro Christian Medical University College, Moshi, Tanzania

## Abstract

**Background:**

Alcohol can be harmful to the development of the foetus. In most developed countries, pregnant women are recommended to abstain from alcohol, however in developing countries, women are less likely to receive these recommendations. With respect to pregnant women in Northern Tanzania, this study aims to 1) describe time trends in level of alcohol consumption, 2) assess socio-demographic predictors of alcohol consumption, and 3) describe associations between alcohol consumption and health-related maternal and foetal outcomes.

**Methods:**

Data related to 34,090 births between 2000 and 2010 was obtained from the Medical Birth Registry at Kilimanjaro Christian Medical Centre (KCMC) in Moshi, Tanzania and analysed. Poisson regression analysis was used to assess associations between potential risk factors and alcohol consumption, and between alcohol consumption during pregnancy and maternal and foetal health outcomes.

**Results:**

From 2000 to 2010, the proportion of women reporting alcohol consumption during pregnancy decreased from 49.5 to 21.5 %. The socio-demographic predictors most strongly related to alcohol consumption were religion (Catholics 53.6 %, Protestants 25.9 %, Muslims 14.8 %) and tribe (Chaggas 45.2 %, Pares 17.3 %, Maasais 6.6 %). Pregnant women consuming alcohol were more likely to be older, taller, and have higher pre-pregnancy body mass index, and were less likely to present with anaemia (Hb < 11.0 g/dl) at last antenatal care (ANC) visit/at admission; adjusted relative risk (ARR) 0.84 (95 % confidence interval 0.79–0.90) for alcohol consumption vs. abstinence. Maternal alcohol consumption during pregnancy was associated with a decreased risk of being small for gestational age (ARR 0.87 (0.80–0.94) and a decreased risk of gestational age less than 37 weeks (ARR 0.89 (0.81–0.99).

**Conclusions:**

The proportion of pregnant women reporting alcohol consumption decreased by 56.5 % from 2000 to 2010. Alcohol intake was strongly associated with socio-demographic factors. The association between alcohol intake and favourable perinatal outcomes remained significant after maternal factors were adjusted for. Information on diet, lifestyle factors and maternal health might give further insight into this unexpected observation. The proportion of pregnant women consuming alcohol in Northern Tanzania is high, and greater awareness of health outcomes associated with alcohol consumption is advised.

## Background

The effect of alcohol consumption on behaviour, health, and society are major public health challenges worldwide [1]. Alcohol consumption during pregnancy generally increases the mother’s risk of a wide range of diseases and affects foetal development negatively. Foetal Alcohol Syndrome (FAS) is the most serious condition caused by the consumption of large amounts of alcohol during pregnancy [2]. Binge drinking is particularly damaging to foetal development [3]. Other consequences for children exposed to alcohol during pregnancy include reduced attention span, receptive language and visual-motor skills [4].

Due to the risks associated with alcohol consumption during pregnancy, pregnant women in many developed countries are recommended to abstain from alcohol. However, even in these countries, formal recommendations to abstain from alcohol during pregnancy are relatively recent: In the United States, the Surgeon General first made the recommendation in 2005 [5].

In contrast to developed countries, policies in many developing countries have paid little attention to risks associated with alcohol consumption during pregnancy. While the alcohol-attributable fraction of the total burden of disease in many developing countries, such as Tanzania, is considerably lower than in Europe and the United States [6], the absolute contribution of alcohol to poor health may be even larger [1]. Although the burden of alcohol use during pregnancy has significant consequences, and at least in some African countries alcohol use among pregnant women is high [7], significant variation exist both within and among African countries[8].

This study aims to:

1. Describe time trends in the level of alcohol consumption over a decade among pregnant women in Northern Tanzania.
2. Assess the association of maternal socio-demographic and pre-pregnancy health factors with alcohol consumption during pregnancy.
3. Assess the relationship between alcohol consumption during pregnancy and maternal and foetal health outcomes.

## Methods

### Study design and data collection

This cross sectional study is based on data from the Birth Registry of Kilimanjaro Christian Medical Centre (KCMC) in Moshi, Kilimanjaro Region in Northern Tanzania. The population in Kilimanjaro Region was approximately 1.6 million people in 2012 [9]. KCMC is a referral hospital that receives patients mostly from Kilimanjaro Region (89.6 %), but also from other regions with only lower level hospitals if more advanced treatment is required. The annual number of births at KCMC is approximately 3,200. Since July 2000 all births at KCMC have been registered in the electronic birth registry file. As of November 2010, when the latest data included in this study was recorded, over 34,000 newborns had been included in the registry. The registry is a collaborative project between KCMC, Kilimanjaro Christian Medical University College, and the University of Bergen, Norway.

Mothers who give birth at KCMC are asked to bring their antenatal care (ANC) card (record of ANC visits during pregnancy) to the hospital. If the mother gives oral consent to participate, a project midwife interviews her with a structured questionnaire within 24 hours after delivery. The topics in the questionnaire include: information concerning the child’s mother and father, living conditions during pregnancy, the mother’s health before and during pregnancy, information concerning the delivery, and on the health of the child/children. Information is also collected from the ANC card.

### Study population

In the analyses of time trends and the relationship between socio-demographic factors/maternal health and alcohol intake during pregnancy, one record per pregnancy was included (*N* = 33,006). In the analyses of the relationship between alcohol intake and perinatal death and low Apgar score, all newborns (including multiple births) were included (*N* = 33,971), but stillbirths were excluded from the analyses of low Apgar score. In the analyses of weight and gestational age, multiple births were excluded as these often are delivered before term and are small for gestational age when compared to singleton standards (*N* = 29,399). Based on a subset of the study population where information on anaemia at last ANC visit or at admission was available, an analysis of anaemia and alcohol consumption during pregnancy was included because anaemia during pregnancy could indicate maternal nutritional status and is a risk factor for poor perinatal outcomes [10]. This information was available for 8,778 pregnancies from 2006 and onwards. In accordance with World Health Organization (WHO) definitions, anaemia was categorized as mild/moderate (Hb 7–10.9 g/dl) or severe (Hb < 7 g/dl) [11].

### Selection and description of risk factors and outcomes

In describing time trends and socio-demographic predictors of alcohol use, the main outcome was the mothers’ drinking habits during pregnancy, dichotomized into alcohol consumption (yes/no) or according to frequency of alcohol consumption. In the multivariable analysis, only socio-demographic and pre-pregnancy health predictors of alcohol consumption with p-values below 0.05 are presented. Among factors that were not significant in the multivariable analysis were marital status and education level of the mother and father.

The association of alcohol with foetal and maternal health outcomes with an occurrence of at least 0.5 % in the registry were considered. Crude and adjusted associations between alcohol consumption and the following health outcomes are presented: maternal anaemia, perinatal mortality, being small for gestational age (below 10th percentile related to gestational age and sex), gestational age below 37 weeks, and low Apgar score 5 minutes after birth. Definitions of health outcomes were selected based on available literature [11–13]. Pregnancy outcomes considered, but not presented due to the non-significant relationship between them and alcohol consumption in the unadjusted analysis, include caesarean section, malformations, head circumference and if the child was transferred to paediatric department postnatally.

### Data analysis

Generalized Linear Models with Poisson regression were used to calculate *p*-values and adjusted relative risks (ARR) with 95 % confidence intervals. In the multivariable models predicting alcohol consumption, only significant factors (*p* < 0.05) were included. In the final model, non-significant factors (*p* > 0.05) were added one by one to assess their significance. All factors were treated as categorical variables in the analysis. All calculations were made with The Statistical Package for Social Science (SPSS) Version 19.0–22.0.

### Ethical approval

The birth registry at KCMC has ethical clearance from the Tanzanian Ministry of Health and National Institute for Medical Research (NIMR), from the Norwegian National Ethics Committee and from the KCM University College. The study is solely based on the KCMC Birth Registry established as a collaborative project between the University of Bergen, Norway and KCMC. The study was initiated in 2011 and approved by birth registry coordinator Rachel Manongi, KCMC, and professor Rolv Terje Lie, University of Bergen, who was the Norwegian project leader for the birth registry project.

## Results

### Overall

34.1 % of mothers reported alcohol consumption during pregnancy, 19.2 % drank occasionally, 3.9 % drank once a week, 10.1 % drank more than once a week and 1.0 % drank alcohol daily.

### Trends

**Fig. 1:**
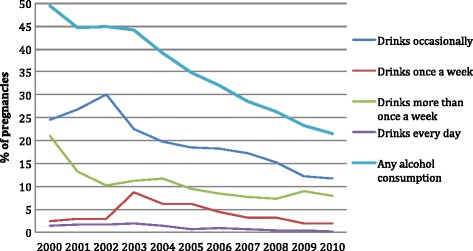
Time trends in alcohol consumption overall and by amount of alcohol consumed during pregnancy

The percentage of mothers who reported alcohol consumption during pregnancy declined from 49.5 % in 2000 to 21.5 % in 2010 (*p* < 0.001) (Fig. 1). A decline was observed in all socio-demographic groups. It was most pronounced in mothers aged 17 or younger, where the decrease was from 29.4 % in 2000 to 6.4 % in 2010. In the oldest mothers, the decrease was from 55.7 % to 33.8 % during the same time period. Frequency of drinking decreased during the observation period: for example, the proportion of women who drank daily declined from 1.4 % in 2000 to 0.1 % in 2010 (*p* < 0.001).

### Socio-demographic and pre-pregnancy health predictors of alcohol consumption

Alcohol consumption varied with most socio-demographic variables (Table 1). Alcohol consumption increased with maternal age, from 17.4 % among mothers aged 17 or younger to 42.5 % among mothers aged 36 years or older. In terms of birth order, 26.7 % of nulliparous women reported to drink during pregnancy compared to 41.1 % of mothers with 3 or more previous pregnancies. 45.2 % of women of the Chagga tribe reported alcohol consumption, which was higher than what women of the Pare tribe (17.3 %), the Maasai tribe (6.6 %) and other tribes (21.8 %) reported. Compared to Protestant and Muslim women, frequency of alcohol consumption during pregnancy was highest amongst Catholic women. Overweight and obese women, tall women and those with positive HIV status were more likely to consume alcohol during pregnancy compared to underweight women and those with a normal weight, short women and those with negative HIV status.

**Table 1:** Socio-demographic and pre-pregnancy health related predictors of alcohol consumption among pregnancies in Northern Tanzania

	% of pregnancies	Mothers who drank alcohol during the pregnancy				
		% drinking alcohol	Univariate analysis		Multivariable model^(a)^	
Variables			RR (95 % CI)	*p*-value	ARR (95 % CI)	*p*-value
Total (*n* = 33006)^(b)^	100.0	34.1				
Mother's age in years (*n* = 32985)				< 0.001		0.001
17 or younger	2.6	17.4	0.41 (0.35 – 0.48)		0.83 (0.66 – 1.03)	
18 – 25	38.9	28.3	0.67 (0.64 – 0.70)		0.93 (0.86 – 1.00)	
26 – 35	48.2	37.9	0.89 (0.85 – 0.93)		1.02 (0.97 – 1.08)	
36 or older	10.3	42.5	1.00		1.00	
Number of previous pregnancies (*n* = 32029)				< 0.001		< 0.001
0	37.0	26.7	0.65 (0.62 – 0.68)		0.82 (0.77 – 0.87)	
1	26.4	36.2	0.88 (0.84 – 0.92)		1.00 (0.95 – 1.05)	
2	17.3	41.8	1.02 (0.97 – 1.06)		1.09 (1.04 – 1.15)	
3 or more	19.3	41.1	1.00		1.00	
Mother's tribe (*n* = 32961)				< 0.001		< 0.001
Chagga	55.8	45.2	2.07 (1.99 – 2.16)		1.34 (1.27 – 1.41)	
Pare	12.6	17.3	0.79 (0.74 – 0.86)		0.92 (0.84 – 1.01)	
Maasai	1.0	6.6	0.31 (0.21 – 0.45)		0.88 (0.57 – 1.35)	
Other	30.6	21.8	1.00		1.00	
Mother's religion (*n* = 32783)				< 0.001		< 0.001
Catholic	38.6	53.6	3.62 (3.41 – 3.84)		2.91 (2.69 – 3.14)	
Protestant	40.0	25.9	1.75 (1.64 – 1.87)		1.59 (1.47 – 1.72)	
Muslim	21.4	14.8	1.00		1.00	
Mother's Body Mass Index before pregnancy (*n* = 25417)				< 0.001		< 0.001
< 18.5 Underweight	5.2	23.8	0.58 (0.50 – 0.67)		0.71 (0.62 - 0.82)	
18.5 – 24.9 Normal weight	56.8	33.7	0.82 (0.73 – 0.91)		0.86 (0.77 - 0.96)	
25.0 – 29.9 Overweight	26.9	41.5	1.01 (0.90 – 1.13)		0.95 (0.85 - 1.06)	
30.0 – 34.9 Obese Class 1 (Moderately Obese)	9.3	44.3	1.07 (0.95 – 1.21)		0.97 (0.87 - 1.09)	
≥ 35.0 Obese Class 2 (Severely Obese)	1.8	41.3	1.00		1.00	
Mother's height (*n* = 29314)				< 0.001		0.001
< 150 cm	5.5	26.7	0.67 (0.60 – 0.73)		0.81 (0.73 – 0.90)	
150 – 159 cm	41.3	33.8	0.84 (0.79 – 0.89)		0.92 (0.86 – 0.97)	
160 – 169 cm	45.9	36.8	0.92 (0.87 – 0.97)		0.91 (0.86 – 0.97)	
≥170 cm	7.3	40.2	1.00		1.00	
Current residence (*n* = 32913)				< 0.001		0.001
Urban area	52.2	34.4	1.00 (0.97 – 1.04)		1.07 (1.03 – 1.11)	
Semiurban area	4.8	28.5	0.83 (0.77 – 0.90)		1.01 (0.92 – 1.11)	
Rural area	43.0	34.3	1.00		1.00	
HIV status (*n* = 33006)				< 0.001		< 0.001
Negative	59.4	28.9	0.69 (0.67 – 0.71)		0.91 (0.87 – 0.95)	
Positive	3.9	39.3	0.94 (0.87 – 1.00)		1.11 (1.01 – 1.21)	
Unknown	36.7	42.0	1.00		1.00	
Father's age (*n* = 32745)				< 0.001		< 0.001
25 or younger	15.1	25.7	0.60 (0.56 – 0.65)		0.82 (0.74 – 0.91)	
26 – 35	55.2	33.0	0.77 (0.73 – 0.83)		0.85 (0.79 – 0.92)	
36 – 45	25.6	40.5	0.95 (0.89 – 1.02)		0.93 (0.86 – 1.00)	
46 or older	4.2	42.6	1.00		1.00	
Father's tribe (*n* = 32835)				< 0.001		< 0.001
Chagga	50.3	45.3	1.85 (1.79 – 1.92)		1.24 (1.19 – 1.30)	
Pare	12.5	19.2	0.78 (0.73 – 0.84)		0.94 (0.87 – 1.03)	
Maasai	1.4	13.6	0.56 (0.44 – 0.70)		1.00 (0.78 – 1.30)	
Other	35.8	24.5	1.00		1.00	
Year (*n* = 33006)				< 0.001		< 0.001
2000	4.8	49.5	2.30 (2.12 – 2.49)		1.88 (1.68 – 2.11)	
2001	10.1	44.7	2.08 (1.93 – 2.24)		1.86 (1.68 – 2.07)	
2002	9.1	44.9	2.09 (1.94 – 2.25)		1.86 (1.68 – 2.07)	
2003	8.1	44.2	2.06 (1.90 – 2.22)		1.72 (1.55 – 1.91)	
2004	8.5	39.1	1.82 (1.68 – 1.97)		1.58 (1.42 – 1.76)	
2005	8.6	34.9	1.62 (1.49 – 1.76)		1.49 (1.34 – 1.66)	
2006	8.9	32.0	1.49 (1.37 – 1.62)		1.46 (1.31 – 1.62)	
2007	9.8	28.5	1.33 (1.22 – 1.44)		1.25 (1.12 – 1.40)	
2008	10.9	26.2	1.22 (1.12 – 1.33)		1.20 (1.08 – 1.34)	
2009	10.8	23.3	1.08 (0.99 – 1.18)		1.02 (0.92 – 1.15)	
2010	10.4	21.5	1.00		1.00	

^(a)^In the multivariable analyses all predictors have been adjusted for mother's age, mother's occupation, mother's tribe, mother's religion, mother's body mass index, mother's height, previous pregnancies, mother's current residence, mother's childhood residence, father's age, father's tribe, year of delivery, drinking water source, boiling of drinking water, mother's age at marriage and mother's HIV-status

^(b)^ For all the predictors one data entry per pregnancy has been included

### Alcohol and anaemia

In the multivariable analyses, the ARR of anaemia (Hb < 11.0 g/dL) was 0.84 (0.79 – 0.90) for those who consumed alcohol compared to those who abstained from alcohol. Corresponding numbers for severe anaemia and mild/moderate anaemia were 0.74 (0.58 – 0.95) and 0.86 (0.80 – 0.93), respectively (Table 2).

**Table 2:** Associations between pregnant women’s alcohol consumption and maternal anaemia in Northern Tanzania 2006–2010

	% of pregnancies	% of pregnancies associated with anaemia	Univariate analysis		Multivariable model^(a)^	
Variables			RR (95 % CI)	*p*-value	ARR (95 % CI)	*p*-value
Anaemia during pregnancy (Hb < 11.0 g/dL) (*n* = 8778)^(b)^						
Drinks alcohol	26.7	46.5	0.81 (0.77 – 0.85)	< 0.001	0.84 (0.79 – 0.90)	< 0.001
Drinks alcohol every day	0.4	43.6	0.76 (0.53 – 1.08)	0.126	0.82 (0.49 – 1.36)	0.439
Drinks alcohol more than once a week	7.4	41.6	0.72 (0.66 – 0.79)	< 0.001	0.77 (0.68 – 0.87)	< 0.001
Drinks alcohol once a week	2.9	44.0	0.77 (0.66 – 0.88)	< 0.001	0.78 (0.64 – 0.95)	0.012
Drinks occasionally	16.0	49.2	0.85 (0.81 – 0.90)	< 0.001	0.88 (0.82 – 0.95)	0.002
Abstains from alcohol	73.3	57.6	1.00		1.00	
Severe anaemia during pregnancy (Hb < 7.0 g/dL) (*n* = 8778)^(b)^						
Drinks alcohol	26.7	6.1	0.75 (0.63 – 0.90)	0.002	0.74 (0.58 – 0.95)	0.016
Drinks alcohol every day	0.4	12.8	1.58 (0.69 – 3.60)	0.276	1.12 (0.44 – 2.86)	0.811
Drinks alcohol more than once a week	7.4	6.0	0.74 (0.54 – 1.02)	0.062	0.51 (0.32 – 0.80)	0.003
Drinks alcohol once a week	2.9	6.3	0.78 (0.48 – 1.27)	0.318	0.70 (0.34 – 1.41)	0.318
Drinks occasionally	16.0	5.9	0.73 (0.58 – 0.91)	0.006	0.87 (0.66 – 1.16)	0.353
Abstains from alcohol	73.3	8.1	1.00		1.00	
Mild/moderate anaemia during pregnancy (Hb 7.0 – 10.9 g/dL) (*n* = 8778)^(b)^						
Drinks alcohol	26.7	40.4	0.82 (0.77 – 0.86)	< 0.001	0.86 (0.80 – 0.93)	< 0.001
Drinks alcohol every day	0.4	30.8	0.62 (0.39 – 1.00)	0.048	0.70 (0.35 – 1.39)	0.310
Drinks alcohol more than once a week	7.4	35.6	0.72 (0.65 – 0.80)	< 0.001	0.82 (0.72 – 0.94)	0.004
Drinks alcohol once a week	2.9	37.7	0.76 (0.65 – 0.89)	0.001	0.79 (0.64 – 0.98)	0.034
Drinks occasionally	16.0	43.3	0.88 (0.82 – 0.93)	< 0.001	0.89 (0.81 – 0.97)	0.007
Abstains from alcohol	73.3	49.5	1.00		1.00	

^(a)^In the multivariable analyses the associations with alcohol have for all the outcomes been adjusted for the following predictors: mother's age, mother's occupation, mother's tribe, mother's religion, mother's body mass index, mother's height, previous pregnancies, mother's current residence, mother's childhood residence, father's age, father's tribe, year of delivery, drinking water source, boiling of drinking water, mother's age at marriage and mother's HIV-status

^(b)^For anaemia during pregnancy, mild/moderate anaemia and severe anaemia one data entry per pregnancy has been included. Results from anaemia, mild/moderate anaemia and severe anaemia are based on 8778 pregnancies registered from 2006 and onwards

### Foetal outcomes

Mothers who reported alcohol consumption during pregnancy were less likely to have babies small for gestational age (below 10th percentile when related to gestational age and sex) and to deliver before term (< 37 weeks) compared with mothers who abstained from alcohol. ARRs were 0.87 (0.80 – 0.94) for delivering a baby that was small for gestational age and 0.89 (0.81 – 0.99) for preterm birth (Table 3), respectively.

**Table 3:** Associations between mothers' alcohol consumption and foetal health outcomes in Northern Tanzania 2000–2010

	% of births	% (*n*) of births with outcome	Univariate analysis		Multivariable model^(a)^	
Variables			RR (95 % CI)	*p*-value	ARR (95 % CI)	*p*-value
Perinatal death (*n* = 33971)^(b)^						
Drinks alcohol	34.1	5.2 (606)	0.83 (0.76 – 0.91)	< 0.001	0.93 (0.81 – 1.07)	0.288
Drinks alcohol every day	1.0	3.3 (11)	0.53 (0.30 – 0.95)	0.032	0.40 (0.17 – 0.96)	0.041
Drinks alcohol more than once a week	10.1	4.6 (157)	0.72 (0.62 – 0.85)	<0.001	0.81 (0.64 – 1.01)	0.064
Drinks alcohol once a week	3.8	5.1 (66)	0.80 (0.63 – 1.02)	0.073	1.05 (0.77 – 1.44)	0.743
Drinks occasionally	19.2	5.7 (372)	0.90 (0.81 – 1.01)	0.071	0.99 (0.85 – 1.16)	0.930
Abstains from alcohol	65.9	6.3 (1414)	1.00		1.00	
Small for gestational age (below 10th percentile related to gestational age and sex^(d)^) (*n* = 27065)^(c)^						
Drinks alcohol	34.4	14.0 (1305)	0.81 (0.76 – 0.86)	< 0.001	0.87 (0.80 – 0.94)	0.001
Drinks alcohol every day	1.0	13.8 (36)	0.80 (0.59 – 1.08)	0.142	0.81 (0.54 – 1.22)	0.316
Drinks alcohol more than once a week	10.0	13.5 (367)	0.78 (0.71 – 0.86)	< 0.001	0.88 (0.77 – 1.00)	0.052
Drinks alcohol once a week	4.0	14.2 (153)	0.82 (0.70 – 0.95)	0.009	0.80 (0.65 – 0.98)	0.034
Drinks occasionally	19.4	14.3 (749)	0.82 (0.77 – 0.89)	< 0.001	0.88 (0.79 – 0.97)	0.007
Abstains from alcohol	65.6	17.3 (3079)	1.00		1.00	
Low gestational age < 37 weeks (*n* = 29399)^(c)^						
Drinks alcohol	34.4	10.5 (1062)	0.85 (0.79 – 0.91)	< 0.001	0.89 (0.81 – 0.99)	0.024
Drinks alcohol every day	1.0	11.7 (33)	0.95 (0.69 – 1.31)	0.735	0.93 (0.61 – 1.41)	0.723
Drinks alcohol more than once a week	10.1	10.1 (301)	0.82 (0.73 – 0.92)	< 0.001	0.90 (0.77 – 1.05)	0.170
Drinks alcohol once a week	4.0	10.5 (123)	0.85 (0.71 – 1.00)	0.055	0.91 (0.72 – 1.15)	0.450
Drinks occasionally	19.4	10.6 (605)	0.86 (0.79 – 0.93)	< 0.001	0.88 (0.78 – 1.00)	0.043
Abstains from alcohol	65.6	12.4 (2394)	1.00		1.00	
Low Apgar score 5 min after birth (Apgar score < 7) (*n* = 32363)^(b)^						
Drinks alcohol	34.2	2.7 (304)	0.87 (0.76 – 0.99)	0.039	1.12 (0.92 – 1.36)	0.249
Drinks alcohol every day	1.0	2.2 (7)	0.70 (0.34 – 1.47)	0.348	0.48 (0.12 – 1.94)	0.300
Drinks alcohol more than once a week	10.2	2.6 (85)	0.82 (0.65 – 1.02)	0.076	1.28 (0.95 – 1.73)	0.104
Drinks alcohol once a week	3.8	2.3 (29)	0.74 (0.51 – 1.06)	0.103	1.23 (0.78 – 1.95)	0.371
Drinks occasionally	19.2	2.9 (183)	0.93 (0.79 – 1.09)	0.384	1.10 (0.88 – 1.38)	0.386
Abstains from alcohol	65.8	3.2 (673)	1.00		1.00	

^(a)^In the multivariable analyses the associations with alcohol have for all the outcomes been adjusted for the following predictors: mother's age, mother's occupation, mother's tribe, mother's religion, mother's body mass index, mother's height, previous pregnancies, mother's current residence, mother's childhood residence, father's age, father's tribe, year of delivery, drinking water source, boiling of drinking water, mother's age at marriage and mother's HIV-status

^(b)^Analyses of perinatal death and low Apgar score 5 min after birth includes all births including twins and triplets. Low Apgar score does not include stillbirths

^(c)^Analyses of small for gestational age and low gestational age include singleton births only

^(d)^Limits for 10th percentile related to gestational age and sex are based on Villar et al., 2014

## Discussion

In this registry-based study of 34,090 deliveries in Northern Tanzania from 2000 to 2010, 34.1 % of pregnant women reported alcohol consumption during pregnancy, with a decline from 49.5 % in 2000 to 21.5 % in 2010. Alcohol consumption was associated with socio-demographic predictors including tribe, age of the mother and father, religion and the mother’s pre-pregnancy body mass index. In general, and contrary to expectations, women who reported alcohol consumption had more favourable pregnancy outcomes than women who abstained from alcohol. These associations were attenuated in the adjusted analysis, although still significant for the outcomes small for gestational age and low gestational age. Similarly, in the sub-study of women with known haemoglobin status (26.5 % of the deliveries), those who consumed alcohol had a *lower* risk of presenting with anaemia at last ANC visit/at admission.

### Drinking pattern and time trends

The proportion of pregnant women in this study who consumed alcohol is higher than what is reported in developed countries. In an American survey from 2006 to 2010, 7.6 % of pregnant women reported alcohol consumption [14]. The proportion of women who abstained from alcohol (65.9 %) in our study is consistent with the WHO’s estimates of lifetime abstainers in Sub-Saharan Africa (65.2 %) [1]. However, regional variations in consumption may be large, and the Kilimanjaro Region has one of the highest reported levels of alcohol consumption per capita in Tanzania [15]. Cultural habits and lack of knowledge about the effect of alcohol on maternal and perinatal health are potential explanations for the high proportion of pregnant women who consume alcohol. According to project midwives at KCMC [personal communication], pregnant women in their district do not routinely receive information about the potentially damaging effects of alcohol on perinatal health. It is therefore likely that during pregnancy, women proceed with drinking the same level of alcohol consumption as before pregnancy, with the exception of women who changed their eating and drinking habits due to nausea.

Notably, from 2000 to 2010, there was a decline in reported alcohol consumption during pregnancy from 49.5 % to 21.5 %. A decline was observed in all socio-demographic groups, although the decline was strongest in younger women. The overall trend was slightly attenuated after adjustment for socio-demographic factors, indicating that a part of the decline was due to changes in the distribution of socio-demographic factors. During ANC visits, the availability of information about the consequences of alcohol consumption on maternal and perinatal outcomes is insufficient; therefore, the observed decline in alcohol consumption is not likely due to knowledge gained during ANC visits. However, a possible explanation for the decline in consumption could still be increased knowledge of the effects of alcohol in the general community. An alternative explanation could be a decrease in financial resources; this could explain the decrease in consumption among the youngest mothers, as it often is the youngest who have the smallest financial resources. Despite growth in the Tanzanian GNP, the poverty rate has remained stable since 2001 at about 30 % of the population [16]. Finally, a changing drinking culture may be another explanation for the decrease in alcohol consumption. In conclusion, a combination of cultural, knowledge-based and economic reasons offer possible explanations for the decline in alcohol consumption among pregnant women in Northern Tanzania.

### Socio-demographic factors

Religion and tribe were the most important predictors of alcohol consumption. This may be due to cultural differences, economy, or traditional food and beverages unique to the area. In Tanzania, where home brew represent about 90 % of the alcohol consumed [17], traditions play a strong role in alcohol consumption. The Chagga tribe are traditionally farmers growing bananas and other crops. Their traditional brew, mbege, is made of bananas and finger millet flowers. Compared to the Chaggas, the Maasai tribe who traditionally are nomads with cattle does not have a strong drinking culture, because traditionally, they did not grow any crops that could be used for brewing. In addition, Maasai women traditionally were not allowed to drink alcohol before a certain age, at which point they drank only on specific occasions. These differences may explain why Chagga women were almost 7 times more likely to drink alcohol as Maasai women during pregnancy.

The results show a strong link between religion and alcohol consumption. Muslims, who are supposed to abstain from alcohol, have the lowest alcohol consumption rates among religious groups. The differences between the two included Christian groups (Catholics and Protestants) may be explained by different views on alcohol, as many Protestant churches, especially Evangelical churches, have been a part of the Temperance or Teetotalism movement [18], while most parts of the Catholic Church have not [19].

### Foetal and maternal outcomes

#### Overall

Mothers who consumed alcohol during pregnancy had newborns with *lower* risks of perinatal death, being small for gestational age, low gestational age and low Apgar score 5 minutes after birth in the crude models. In the multivariable models, the lower risks of being small for gestational age and low gestational age for children born to mothers who consumed alcohol during pregnancy remained statistically significant. Previous studies have shown various results of low/moderate alcohol consumption during pregnancy, with some studies reporting protective associations with respect to stillbirth, intrauterine growth restriction, low birth weight, and preterm delivery [20–23]. In 2007 a metaanalysis of data from mainly developed countries was inconclusive about maternal alcohol consumption’s effect on foetal health [24]. Hence, unmeasured confounders, such as beneficial nutrients in traditional alcoholic beverages, or even a positive effect of alcohol per se are possible explanations for these findings.

### Diet and maternal health

Although the results are adjusted for maternal height, weight and many socio-demographic factors, there may still be unmeasued confounding variables. In many developing countries, the most important predictors of health are income, education and occupation [25]. Data used for this study contains no information on personal income. Women who are able to purchase alcohol may have more economic resources, and consequently, more and better food. This would positively affect their daily caloric intake, which is the most important nutritional factor determining birth weight [26]. In addition there may be a “healthy drinker” effect where mothers with a poor obstetric history choose to abstain from alcohol [24].

### Nutrients in traditional brew

Another possible explanation for why mothers who consumed alcohol during pregnancy had healthier newborns is that there are substances in traditional alcoholic beverages that are beneficial for the foetus. In Tanzania, most alcohol consumed is from local brews, but the main ingredients differ from tribe to tribe. The local brew of the Chagga tribe, mbege, is rich in nutrients and traditionally therefore considered healthy. There may be vitamins and minerals in local brews that are beneficial to the foetus, and consumers of traditional beer are reported to have higher folate and vitamine B12 levels [27]. A recent review, however, showed that folate supplementation did not significantly reduce perinatal death, preterm birth or low birth weight [28]. In our study, alcohol was not associated with perinatal death in Chaggas (RR = 0.98) whereas relative risk was 0.83 for other tribes. This may indicate that the effect of alcohol is not related to nutrients in the Chagga brew.

### Iron and anaemia

Pregnant women who consumed alcohol were less likely to present with anaemia at last ANC/at delivery. One explanation for this may be that traditional Tanzanian brews are often cooked in iron pots before fermentation [29]. In contrast, commercially prepared beer lacks iron. Inadequate iron intake is a cause of anaemia and several countries advise pregnant women to take daily iron supplements [30]. Surveys have shown that if the mother takes iron supplements during pregnancy, the baby will have a higher birth weight and a lower risk of being born preterm [31].

### Limitations

The registry file used for these analyses pertained to deliveries at KCMC, where patients may have demographic characteristics that are unique from the rest of the Kilimanjaro Region population. Treatment at KCMC may differ from home deliveries and less equipped hospitals. Hemoglobin was only recorded after 2006. The registry is based on self-reports, which may be unreliable. For example, alcohol consumption is commonly overestimated or underestimated [32] based on local culture and its acceptance of alcohol. In terms of alcohol, only information on consumption frequency exists, not the absolute amounts consumed. This makes it difficult to compare our results to other surveys and predictions of dose–response relationships may become unreliable.

The registry does not contain detailed information on some important aspects of pregnant womens’ health status, such as nutrition level. Compared to developed countries, nutrition in Tanzania is less varied and malnutrition is far more common. As alcohol consumption might be an indicator of the mothers’ nutrition level, nutritional status as a cause of the observed associations cannot be ruled out.

Another limitation is that the birth registry does not collect data on newborn conditions that are not evident at birth. It is therefore not possible to evaluate whether mothers who consumed alcohol during pregnancy have healthier babies in the long term. As alcohol is estimated to be responsible for only 1 % of low birth weight cases in most African countries [6], it is difficult to demonstrate the effect of alcohol without monitoring more prominent causes of low birth weight and of being small for gestational age.

## Conclusions

From 2000 to 2010, the percentage of women who reported alcohol consumption during pregnancy decreased from 49.5 % to 21.5 %. The reason for this decline remains unclear, but a combination of explanations is likely, and include cultural, knowledge-based and economic factors, such as poor economy among young mothers.

The socio-demographic factors that were most strongly related to alcohol consumption were religion and tribe. Mothers with a positive HIV-status drank alcohol more frequently than those with a negative HIV-status. Alcohol intake may interfere with the effect of HIV treatment.

Alcohol was associated with *better* birth outcomes, however, it is unlikely that alcohol consumption in itself can explain these findings. Better nutrition and health among mothers who reported alcohol consumption are more likely reasons for these associations, but our data do not allow for a detailed analysis that would fully account for these factors.

Further studies are needed to assess whether knowledge about the effects of alcohol in Tanzania is changing, and to assess long term effects of alcohol during pregnancy.
